# Efficient and Selective Removal of Heavy Metals and Dyes from Aqueous Solutions Using Guipi Residue-Based Hydrogel

**DOI:** 10.3390/gels10020142

**Published:** 2024-02-13

**Authors:** Xiaochun Yin, Pei Xu, Huiyao Wang

**Affiliations:** Department of Civil Engineering, New Mexico State University, Las Cruces, NM 88003, USA; pxu@nmsu.edu

**Keywords:** hydrogel, cellulose, dyes removal, heavy metals removal, adsorption, waste reutilization

## Abstract

The presence of organic dyes and heavy metal ions in water sources poses a significant threat to human health and the ecosystem. In this study, hydrogel adsorbents for water pollution remediation were synthesized using Guipi residue (GP), a cellulose material from Chinese herbal medicine, and chitosan (CTS) through radical polymerization with acrylamide (AM) and acrylic acid (AA). The characteristics of the hydrogels were analyzed from a physicochemical perspective, and their ability to adsorb was tested using model pollutants such as Pb^2+^, Cd^2+^, Rhodamine B (RhB), and methyl orange (MO). The outcomes revealed that GP/CTS/AA-co-AM, which has improved mechanical attributes, effectively eliminated these pollutants. At a pH of 4.0, a contact duration of 120 min, and an initial concentration of 600 mg/L for Pb^2+^ and 500 mg/L for Cd^2+^, the highest adsorption capabilities were 314.6 mg/g for Pb^2+^ and 289.1 mg/g for Cd^2+^. Regarding the dyes, the GP/CTS/AA-co-AM hydrogel displayed adsorption capacities of 106.4 mg/g for RhB and 94.8 mg/g for MO, maintaining a stable adsorption capacity at different pHs. Compared with other competitive pollutants, GP/CTS/AA-co-AM demonstrated a higher absorption capability, mainly targeted toward Pb^2+^. The adsorption processes for the pollutants conformed to pseudo-second-order kinetics models and adhered to the Langmuir models. Even after undergoing five consecutive adsorption and desorption cycles, the adsorption capacities for heavy metals and dyes remained above 70% and 80%. In summary, this study effectively suggested the potential of the innovative GP/CTS/AA-co-AM hydrogel as a practical and feasible approach for eliminating heavy metals and dyes from water solutions.

## 1. Introduction

Water is a crucial resource for the survival of all living organisms. Nevertheless, due to the swift progress of industrialization, there has been a notable and alarming rise in water pollution, posing an escalating and pressing environmental and public concern. [1] In recent decades, there has been a concerning increase in water pollution caused by various compounds such as heavy metal ions, dyes, and radioactive wastes. It is imperative to prioritize safeguarding water quality [2]. Organic dyes and heavy metal ions pose specific concerns within sewage systems, primarily contributing to contamination [3] Heavy metals, known for their raised toxicity levels, present prominent dangers to aquatic environments and essential human organs like the central nervous system, lungs, gastrointestinal tract, and kidneys [4]. Concerning dyes, their potential to disrupt gas solubility and decrease water clarity persists even at concentrations below 1 mg/L [4]. The resistance to natural degradation exhibited by these chemical pollutants due to their complex and stable structures compounds the challenge of their persistence in the environment, threatening both ecosystems and human well-being [5].It is crucial to take urgent action to address the increasing problem of water pollution and protect the purity of water sources.

Over the past few years, various techniques have been employed to eliminate dyes and heavy metals from water sources, including photocatalytic degradation [6], membrane filtration [7], electrolysis [8], electrodialysis [9], biodegradation [10], precipitation [11], and adsorption [12]. However, there are constraints on the practical application of these purification methods due to economic considerations. The need for effective and affordable technologies has driven ongoing research efforts in water purification. Among the various purification methods, adsorption emerges as a physicochemical separation technique that is both environmentally friendly and highly effective for treating contaminated water [13,14]. This method effectively removes toxic pollutants at low concentrations while remaining economically feasible and producing minimal secondary waste [15]. Furthermore, the materials utilized as adsorbents for separation can be revitalized and employed again, leading to lowered water purification expenses and a reduction in waste production [16]. Yudaev et al. [17] developed a magnetic sorbent incorporating an organophosphorus extractant to extract palladium from hydrochloric acid media in a highly efficient and environmentally friendly manner. Li et al. [18] reported the development of adsorbents based on bifunctional solid-state ionic-liquid-supported amidoxime chitosan (ACS), aiming to simultaneously adsorb two metals from aqueous solutions. In our previous investigations, we demonstrated that adsorbents based on polymers exhibit high efficiency in the removal of heavy metals (e.g., Pb^2+^, Cu^2+^) [12]. These adsorbents also effectively remove dye pollutants from aqueous solutions [19,20]. Therefore, adsorption is an efficient and friendly method for the removal of heavy metals and dyes from aqueous solutions, and substantial attention has been directed towards advancing adsorption materials, encompassing carbon-based hydrogels [21,22].

Hydrogels are becoming a focal point of interest due to their intricate three-dimensional porous architectures, inherent hydrophilicity, and the presence of functional groups (-COOH, -NH_2_, -SO_3_H, -OH) that facilitate the capture of dyes and metal ions from wastewater [23]. Hydrogel, derived from natural polymers such as chitosan, sodium alginate, and cellulose, has garnered substantial attention due to its heightened safety, hydrophilicity, and biocompatibility [24,25]. Hydrogels made from natural polymers have advantages over other materials due to their inherent properties and performance. For example, biochar has limited adsorption capacity [26], while activated carbons lack pollutant selectivity and face challenges in regeneration and recycling [3]. Carbon dots also suffer from intricate separation and recycling issues [27].

Cellulose is a widely available material that makes up a significant part of biomass production. It has been extensively used to create eco-friendly and biocompatible hydrogels for removing heavy metals and dyes from aqueous water [28,29,30]. Nonetheless, a significant quantity of cellulose goes to waste, as numerous agricultural by-products are discarded without fully harnessing their potential applications [1]. In traditional Chinese medicine, the Guimpe Tang (GP) prescription incorporates various ingredients, including Baizhu, Danggui, Baifuling, fried Huangqi, Longan flesh, Yuanzhi, and fried Sour Jujube Kernel. This prescription is extensively utilized to treat medical conditions marked by deficiencies in both Qi and blood in the heart and spleen, giving rise to symptoms such as palpitations, a lack of focus, sleeplessness, excessive sweating at night, sensations of feverishness, and fatigue. The adequacy of cellulose components within waste residues derived from Chinese herbal sources has not been adequately addressed in previous research endeavors [31]. Given the substantial yearly accumulation of waste residue resulting from the Chinese medicine industry, the implementation of effective management techniques becomes imperative to mitigate environmental contamination and foster sustainable waste disposal approaches [32,33]. Hence, the proficient regulation of cellulose content in GP residues presents a significant challenge while concurrently representing an avenue for transforming waste streams into valuable resources for purposeful reutilization.

The utilization of Chinese herb residue has been suggested as a potential method for the production of cellulose-based hydrogels [22]. However, cellulose extraction for environmentally sustainable conversion purposes presents inherent difficulties. This extraction process requires large amounts of chemicals, leading to complex protocols and the production of waste products. Hence, the focal point of this investigation is to maximize the utilization of this residue to enhance overall efficiency. Nevertheless, the utilization of cellulose-based hydrogels in the context of wastewater treatment is hampered by inherent constraints, including restricted selectivity, challenges in regeneration, and compromised mechanical strength [34]. To augment the mechanical characteristics of hydrogels, incorporating natural materials such as chitosan, known for its eco-friendly, biodegradable, and compatible properties, can fortify structural integrity under the influence of alkali/urea and freeze–thaw cycles [35,36]. Consequently, this gives rise to a more resilient hybrid hydrogel derived from cellulose and chitosan, exhibiting enhanced resistance to mechanical compromise and deterioration [37]. Moreover, research has delved into covalent bonding techniques aimed at incorporating hydrophilic functionalities, such as carboxylic acid groups (-COOH) and amino groups (-NH_2_). These functionalities serve to introduce active groups within the hydrogels, thereby augmenting selectivity towards adsorbed pollutants and enhancing the overall adsorption efficiency of the synthesized hydrogels [38,39].

The core objective of this study is to elevate the mechanical attributes, selectivity, and regenerative potential through the synergistic utilization of GP residue and chitosan (CTS) as the principal source materials. This goal is accomplished by employing a meticulously orchestrated process involving alkali/urea treatment facilitated by microwave assistance, coupled with the strategic implementation of free radical polymerization introducing acrylamide (AM) and acrylic acid (AA). The outcome of these integrated techniques is the creation of a novel spectrum of hydrogels rooted in GP residue, thereby marking a significant advancement in this domain. This resulting hydrogel (GP/CTS/AA-co-AM), with the help of CTS, exhibited excellent physicochemical characteristics and higher mechanical strength (0.25 MPa). The hydrogel exhibited exceptional adsorption capabilities towards Pb^2+^, Cd^2+^, Rhodamine (RhB), and methyl orange (MO), demonstrating excellent proficiency in terms of capacity, kinetics, and selectivity. The GP/CTS/AA-co-AM hydrogel consistently exhibited effective adsorption behavior for RhB and MO across a range of pH conditions. This innovation repurposes Chinese herbal residue to create an economical and effective hydrogel adsorbent for eliminating contaminants such as Pb^2+^, Cd^2+^, RhB, and MO, presenting a promising solution for tackling environmental pollution.

## 2. Results and Discussion

### 2.1. Structural Characterization and Property Analysis of the Hydrogels

**Figure 1:**
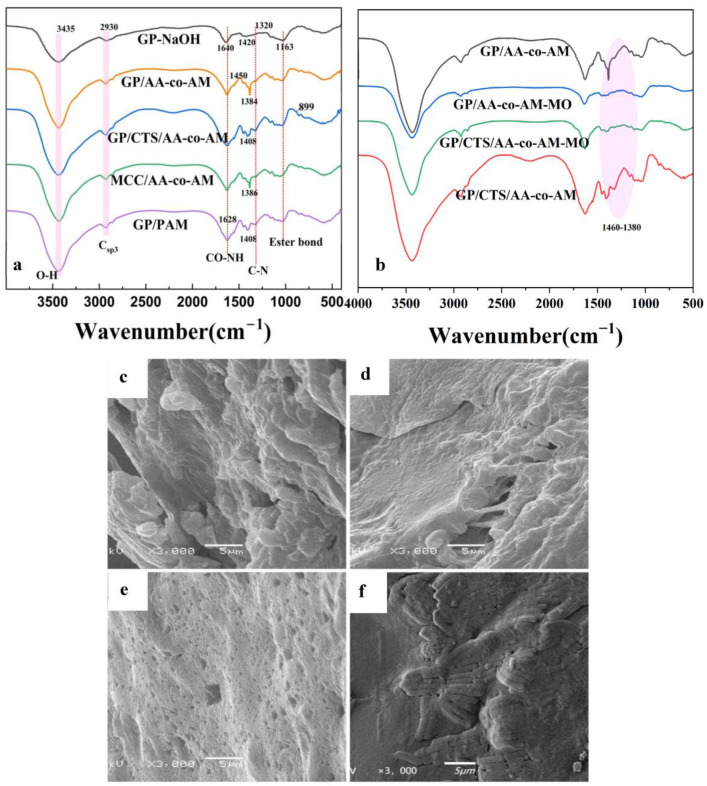
The FTIR of hydrogels before (**a**) and after the adsorption (**b**); SEM of (**c**) GP/AA-co-AM, (**d**) GP/GTS/AA-co-AM, (**e**) GP/PAM, and (**f**) MCC/AA-co-AM.

The FTIR analysis revealed diverse functional groups within the hydrogels, as depicted in Figure 1a. Across all the sample FTIR spectra, the pronounced absorption bands at around 3435 cm^−1^ correspond to the inter- and intramolecular hydrogen-bonded stretching of -OH groups. A distinct peak at 2930 cm^−1^ can be attributed to the presence of Csp3. Additionally, the stretching of CO-NH groups is evident at 1628 cm^−1^ [40], while the absorption peak at 1450 cm^−1^ signifies methylene deformation. The presence of the C-N absorption band (amide III bands) is evident at 1413 cm^−1^, and the peaks at 1163 cm^−1^ indicate the stretching vibration of the ester bond within the cellulose ester group, thereby confirming the retention of characteristic cellulose features within the hydrogels. The peaks at 1384 cm^−1^ align with C-O stretching vibrations. The peak at 899 cm^−1^ emerges from the central expansion vibration of chitosan, thus affirming the presence of distinctive chitosan attributes. In the aggregate, the FTIR spectroscopy outcomes substantiate the successful integration of AA and AM via graft polymerization reactions in the hydrogels. Furthermore, they offer validation of the existence of chitosan within the GP/CTS/AA-co-AM hydrogel.

Figure 1b demonstrates that after the hydrogel underwent dye adsorption, a significant portion of the peaks within the 1380–1460 cm^−1^ range vanished. This disappearance can be attributed to the electrostatic interactions between the dye molecules and the hydrogel. These interactions may induce conformational changes in the polymer chains of the hydrogel or rearrange its molecular structure. Consequently, the FTIR spectra display alterations, signifying modifications in the chemical environment of the functional groups present in the hydrogel. Additionally, the presence of dyes on the hydrogel surface may occupy specific binding sites or alter the local microenvironment around the functional groups, thereby influencing the vibrational frequencies of the chemical bonds. As a result, peaks within the 1380–1460 cm^−1^ range are no longer evident in the FTIR spectra due to the interactions between the adsorbed dyes and the molecular structure of hydrogel.

The surface morphologies and characteristics of the hydrogels were thoroughly examined using SEM, as depicted in Figure 1c–f. The SEM imaging revealed that all the analyzed specimens exhibited a coarse and irregular surface topography marked by distinctive porous formations. This porous microstructure offers benefits by yielding an expanded surface area and abundant adsorption sites for pollutants, consequently augmenting the overall adsorption effectiveness of the hydrogels. Notably, within the hydrogel array, GP/AA-co-AM and GP/CTS/AA-co-AM displayed particularly prominent and irregular porous configurations, suggesting their potential for elevated adsorption capacity and efficacy in comparison to MCC/AA-co-AM and GP/PAM. Furthermore, the architecture of GP/CTS/AA-co-AM displayed heightened density when compared to GP/AA-co-AM due to the incorporation of CTS, resulting in enhanced cross-linking density within the hydrogel structure. Additionally, GP/PAM exhibited the most compact structure among all the investigated hydrogels, implying a relatively lower adsorption capacity than GP/CTS/AA-co-AM, GP/AA-co-AM, and MCC/AA-co-AM. These observations underscore the pivotal role of structural attributes in influencing the adsorption characteristics and performance of hydrogels.

Figure S1a–i depicts the SEM of GP/AA-co-AM and GP/CTS/AA-co-AM, showcasing pronounced alterations in their surface characteristics pre- and post-adsorption of Pb^2+^ and MO. The surface attributes of GP/AA-co-AM and GP/CTS/AA-co-AM prior to the adsorption of dyes manifested discernible cellulose patterns alongside an irregular and uneven surface morphology. This distinctive surface structure improved the proficiency of the hydrogels in efficiently sequestering heavy metal ions and dye molecules. After the adsorption process, the overall surface texture of the hydrogels exhibited increased smoothness, particularly following the incorporation of Pb^2+^. Based on this phenomenon, it seems that the adsorbed entities merged. The hydrogel composition encompasses carbohydrates, lignin, and cellulose, each possessing functional moieties that jointly introduce a diverse array of functional groups originating from the AA and AM, such as -OH, -COOH, and -NH_2_. These constituents facilitated interactions with ligands via mechanisms like electrostatic forces and hydrogen bonding, consequently inducing alterations in the surface morphology of the hydrogels.

**Figure 2:**
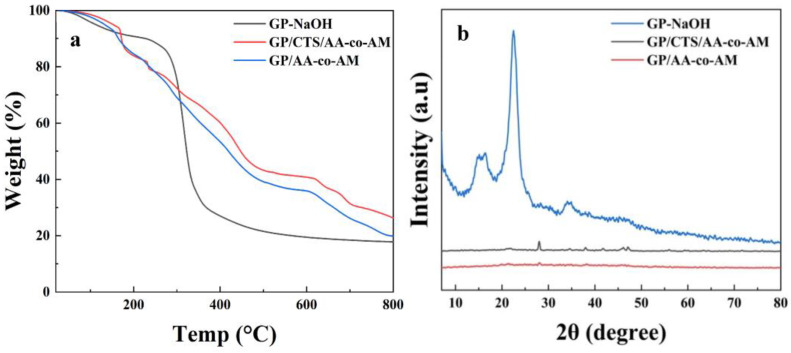
TGA curves (**a**) of GP-NaOH, GP/AA-co-AM, and GP/CTS/AA-co-AM and XRD curves (**b**) of GP-NaOH, GP/AA-co-AM, and GP/CTS/AA-co-AM.

The thermal characteristics of the modified GP-NaOH- and GP-based hydrogels were assessed using TGA, as shown in Figure 2a. The weight loss observed below 100 °C can be attributed to the loss of bound water in the samples [41]. In the case of GP-NaOH, a distinct and rapid decomposition rate was evident in the temperature span of 300 °C to 350 °C, indicating the degradation of cellulose accompanied by a minor residue of carbide. Conversely, in the case of GP/CTS/AA-co-AM and GP/AA-co-AM, the primary degradation phases exhibited extended durations, accompanied by a mitigation in the rate of decomposition. Furthermore, an elevation in the quantity of residual carbide was observed in GP/CTS/AA-co-AM in comparison to GP-NaOH and GP/AA-co-AM. Furthermore, the degradation of carboxyl groups originating from the AA and amide groups derived from the AM transpired within a temperature span of 340 °C to 500 °C [42].

XRD was utilized to analyze the crystalline properties and structural phases of GP-NaOH, GP/CTS/AA-co-AM, and GP/AA-co-AM, as illustrated in Figure 2b. The XRD pattern of GP-NaOH revealed four well-defined diffraction peaks located at 14.8°, 16.4°, 22.5°, and 34.7°, corresponding to the 101, 101’, 002, and 040 crystallographic planes of cellulose in its crystalline state. These results aligned with the crystalline arrangement observed in microcrystalline cellulose (MCC) and prior studies [43]. A sharp peak at 2θ = 22.5°, corresponding to the 002 planes, indicated a typical crystal lattice of cellulose I*ß* [44]. In comparison, the XRD profiles of GP/AA-co-AM and GP/CTS/AA-co-AM exhibited wide and less defined peaks at 2θ = 22° and 2θ = 38°, signifying the amorphous nature of both hydrogel specimens [45]. These observations are in agreement with the findings on hydrogel MCC-g-(AA-co-AM) reported by Zhao et al. [40], suggesting that the crystalline structure of GP-NaOH dissipated upon dissolution, and the inclusion of AA further intensified the amorphous character of the adsorbent [46]. These outcomes substantiate the grafting of cellulose with AA/AM, a conclusion in harmony with the earlier elucidated FTIR results.

The empirical findings demonstrated an augmentation in the mechanical attributes of the hybrid hydrogels through the incorporation of chitosan, as outlined in Table S1. Both the tensile elastic modulus and the compressive modulus showed a substantial elevation compared to the hydrogels devoid of chitosan. The GP/CTS/AA-co-AM hydrogel showcased a compressive modulus of 0.25 MPa and a strain modulus of 0.21 MPa, underscoring its enhanced mechanical prowess when compared to the hydrogel GP/AA-co-AM. This observed elevation in these parameters can be ascribed to the escalated cross-linking density brought about by the introduction of chitosan.

### 2.2. pH-Responsive Swelling Behavior of Hydrogels

**Figure 3:**
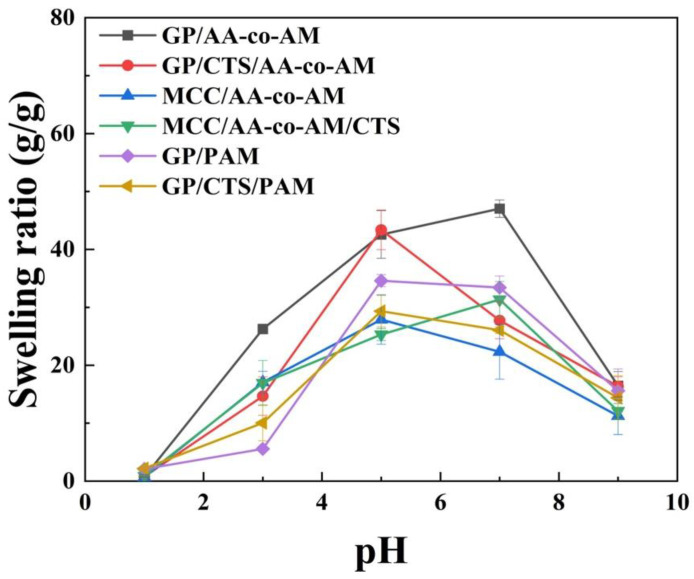
The swelling ratio of the hydrogels at different pHs.

The pH-responsive swelling behavior of the hydrogels was investigated by immersing them in aqueous solutions within a pH range of 1.0 to 9.0, adjusted using 0.1 M of HCl and 0.1 M of NaOH. Figure 3 presents the hydrogel swelling ratio of the samples as a function of pH. It was observed that for GP/CTS/AA-co-AM, MCC/CTS/AA-co-AM, GP/PAM, and GP/CTS/PAM, the swelling ratio displayed an increase as the pH increased from 3.0 to 5.0. These findings elucidate the pH-dependent nature of the swelling behavior of hydrogels. Similarly, for GP/AA-co-AM and MCC/AA-co-AM, a significant increase in the swelling ratio was observed within the pH range of 3.0 to 7.0. However, beyond pHs of 5.0 and 7.0, a significant decline in the swelling ratio was observed. At a pH of 5.0, the highest swelling ratio observed for GP/CTS/AA-co-AM was approximately 42.2 g/g, while for GP/AA-co-AM, the highest swelling ratio reached approximately 48.3 g/g at pH 7.0.

These phenomena can be attributed to the influence of pH on the anion–anion repulsive forces between the -COOH present in the hydrogel network. With an increase in pH, the -COOH undergoes deprotonation, resulting in increased repulsive forces between these negatively charged groups. As a result, the hydrogel network expanded, leading to a higher swelling ratio. However, when the pH was less than 3.0, the -COOH was more protonated, weakening the anion–anion electrostatic repulsive forces and decreasing the hydrogel’s swelling ratio [47]. Furthermore, the addition of CTS to the hydrogels decreased the swelling ratio compared to the hydrogels without CTS, as shown through SEM. This can be attributed to the increased cross-linking density within the hydrogel network induced by CTS. The higher cross-linking density restricted the swelling capacity of the hydrogel, thereby leading to a reduced swelling ratio.

### 2.3. Adsorption Capacity of Hydrogels

#### 2.3.1. Effect of pH on Adsorption 

**Figure 4:**
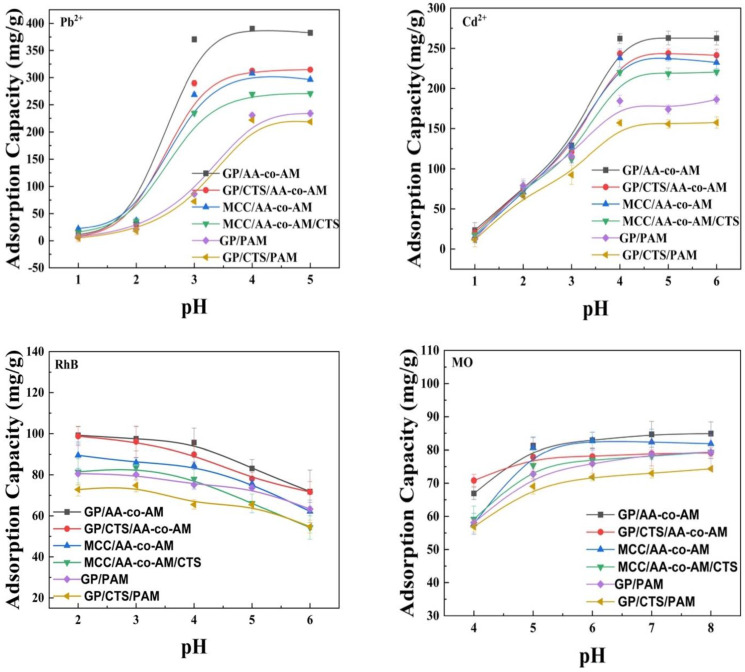
Effects of pH on Pb^2+^, Cd^2+^, RhB, and MO adsorption. Adsorption conditions: temperature, 25 °C; contact time, 150 min; adsorbent, 20 mg; volume of adsorption solution, 50 mL; initial concentration: Pb^2+^, 600 mg/L; Cd^2+^, 500 mg/L; RhB, 200 mg/L; MO, 100 mg/L.

The adsorption characteristics of the hydrogels are subject to modulation by the pH of the solution, as clearly illustrated in Figure 4. Given that the precipitation tendency of heavy metal ions becomes prominent at a pH exceeding 5.0, an in-depth exploration of the adsorption capacity was conducted within a pH range of 1.0–5.0. The adsorption capacity of the hydrogel for Pb^2+^ and Cd^2+^ exhibits an ascending trend alongside elevating pH values, reaching a state of equilibrium around a pH of 4.0. Conversely, the adsorption capacities at pHs below 1.0 for heavy metal ions demonstrate a negligible value, which undergoes a rapid surge as the pH progresses into a range of 2.0 to 4.0. This phenomenon can be attributed to the interplay between H^+^ and metal ions, competing for the available binding sites on the hydrogel surface at a pH below 1.0. As the pH rose, the concentration of hydrogen ions experienced a gradual decline, mitigating the competitive influence on metal ions and consequently augmenting the adsorption capacity. Furthermore, the swelling propensity of the hydrogels experiences a marked reduction at a pH of 1.0, which exerts an additional impact on the adsorption capacity. Subsequent investigative efforts led to determining the optimal pH condition for hydrogel-mediated heavy metal ion adsorption, settling at a pH of 4.0.

The adsorption capacities of hydrogels for heavy metals followed the order of GP/AA-co-AM > GP/CTS/AA/co-AM > MCC/AA-co-AM > MCC/AA-co-AM/CTS > GP/PAM > GP/CTS/PAM. The most significant adsorption capacities were observed at a pH of 4.0 for Pb^2+^ and Cd^2+^ on GP/AA-co-AM, reaching 390.3 mg/g and 262.3 mg/g, respectively. Similarly, GP/CTS/AA-co-AM exhibited considerable adsorption capacities of 312.7 mg/g and 243.4 mg/g for Pb^2+^ and Cd^2+^, respectively. These values surpassed the adsorption capacities of MCC/AA-co-AM (307.6 mg/g for Pb^2+^ and 238.1 mg/g for Cd^2+^) as well as MCC/AA-co-AM/CTS (269.4 mg/g for Pb^2+^ and 220.1 mg/g for Cd^2+^). The disparities in the adsorption capacity between the GP-based hydrogels and the MCC-based hydrogels can be ascribed to the existence of lignin within GP. Lignin functions analogously to amphiphilic cations and has been proposed to attenuate the hydrophobic interactions associated with cellulose, thereby expediting its dissolution [48]. Hence, lignin functions as an eco-friendly additive that amplifies the dissolution of cellulose within aqueous-based cold alkali systems, resulting in the exposure of more active sites, thereby enhancing the adsorption capacities of GP/AA-co-AM and GP/CTS/AA-co-AM hydrogels [49].

The adsorption behavior of RhB and MO dyes, representatives of cationic and anionic organic chemicals, respectively, was thoroughly examined on various hydrogel compositions under different pHs. RhB was investigated within a pH range of 2.0–6.0, while MO was assessed within a pH range of 4.0–8.0. The hydrogels demonstrated higher adsorption capacities for RhB at lower pHs, whereas the adsorption of MO was more pronounced at higher pHs. The hydrogels GP/AA-co-AM and GP/CTS/AA/co-AM displayed the highest recorded adsorption capacities for RhB, measuring 99.2 mg/g and 98.7 mg/g at a pH of 2.0, respectively. Similarly, at a pH of 8.0, GP/AA-co-AM and GP/CTS/AA/co-AM exhibited the highest adsorption capacities for MO, measuring 84.9 mg/g and 79.1 mg/g, respectively. 

However, the adsorption capacities of the hydrogels for both RhB and MO did not undergo significant changes due to the opposing effects of protonation and deprotonation on the -COOH and -NH_2_ groups in the hydrogels. These groups can undergo reversible protonation and deprotonation reactions depending on the pH of the solution. Hence, in contrast to the adsorption of heavy metals, pH alterations did not result in substantial fluctuations in the adsorption capacities of the hydrogels for RhB and MO. Despite the noticeable variations in the swelling ratios of the hydrogels under different pH conditions, the adsorption capacities for the two dyes remained relatively consistent. These observations indicate that the adsorption capacities of the hydrogels exhibit stability under varying pH conditions, rendering them suitable for a broader range of applications.

#### 2.3.2. Effect of Contact Time on Adsorption and Adsorption Kinetics

**Figure 5:**
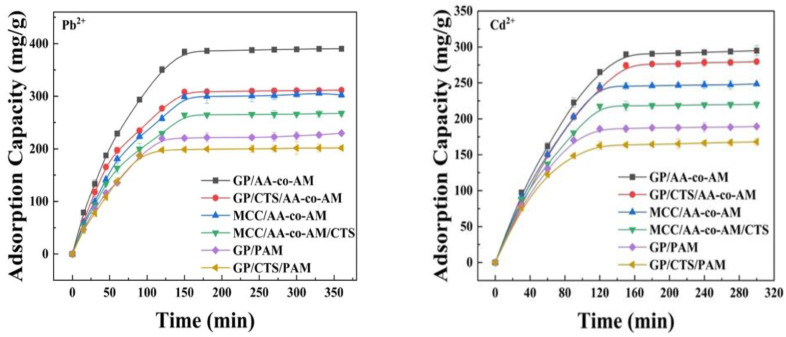

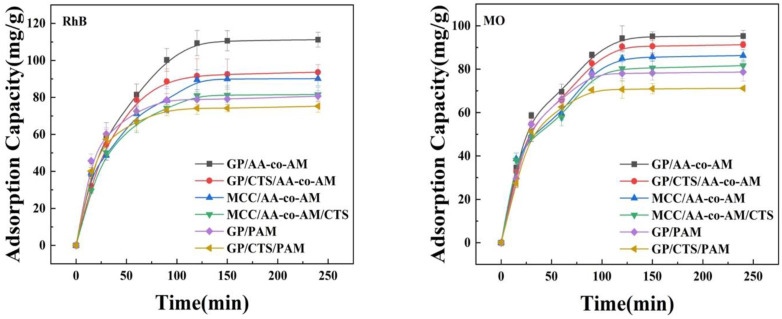
Effects of contact time on hydrogels’ adsorption of Pb^2+^ and Cd^2+^. Adsorption conditions: temperature, 25 °C; adsorbent, 20 mg; volume of adsorption solution, 50 mL; pH, 4.0 for Pb^2+^ and Cd^2+^, 3.0 for RhB, and 8.0 for MO; initial concentration: Pb^2+^, 600 mg/L; Cd^2+^, 500 mg/L; RhB, 200 mg/L; MO, 100 mg/L.

Figure 5 illustrates the impact of contact time on the adsorption performance of the hydrogels. The equilibrium adsorption of Pb^2+^ and Cd^2+^ ions onto the hydrogels was attained within 150 min. Both the GP/AA-co-A-AM and GP/CTS/AA-co-AM hydrogel configurations exhibited expeditious adsorption capacities for the respective heavy metals, with peak adsorption capacities of 384.2 mg/g and 307.9 mg/g for Pb^2+^, and 289.9 mg/g and 274.5 mg/g for Cd^2+^. This phenomenon can be attributed to the interaction between the adsorption sites and the heavy metal ions during the initial phase, spanning 0 to 150 min. Subsequently, the ongoing adsorption of heavy metal ions predominantly transpired at the internal adsorption sites, resulting in a gradual augmentation of the adsorption capacity over the subsequent duration (second phase, 150 to 350 min).

The influence of contact time on the absorption of the RhB and MO dyes in the hydrogel samples within a time range of 15 to 240 min was investigated. It was observed that the adsorption rate of the hydrogels for the dyes exceeded that of the heavy metals. The highest recorded adsorption capacities for RhB were 109.4 mg/g and 92.5 mg/g in the GP/AA-co-AM and GP/CTS/AA-co-AM hydrogels, respectively, at 120 min. Similarly, the highest adsorption capacities for MO were 95.3 mg/g and 90.6 mg/g in the same hydrogel compositions at 120 min. The observed phenomenon of reaching equilibrium within 120 min for both RhB and MO, followed by a stable adsorption capacity, can be attributed to the saturation of the dyes through electrostatic bonding facilitated by the presence of abundant functional sites on the hydrogel surface. Furthermore, the rapid adherence of dye molecules to the three-dimensional structured adsorbents played a significant role. As a substantial number of dye molecules occupied the active sites of hydrogels, the adsorption rate gradually decreased.

To thoroughly explore the adsorption kinetics, we employed the pseudo-first-order and pseudo-second-order kinetic models, as outlined by [22]. The pseudo-first-order model posits that the adsorption pace is predominantly governed by diffusion and mass transfer, whereas the pseudo-second-order model contends that the rate-determining step is driven by chemical adsorption. Subsequently, the kinetic adsorption dataset was subjected to fitting using the pseudo-first-order and pseudo-second-order kinetic models. The corresponding equations can be found in the Supplementary Information.

**Figure 6:**
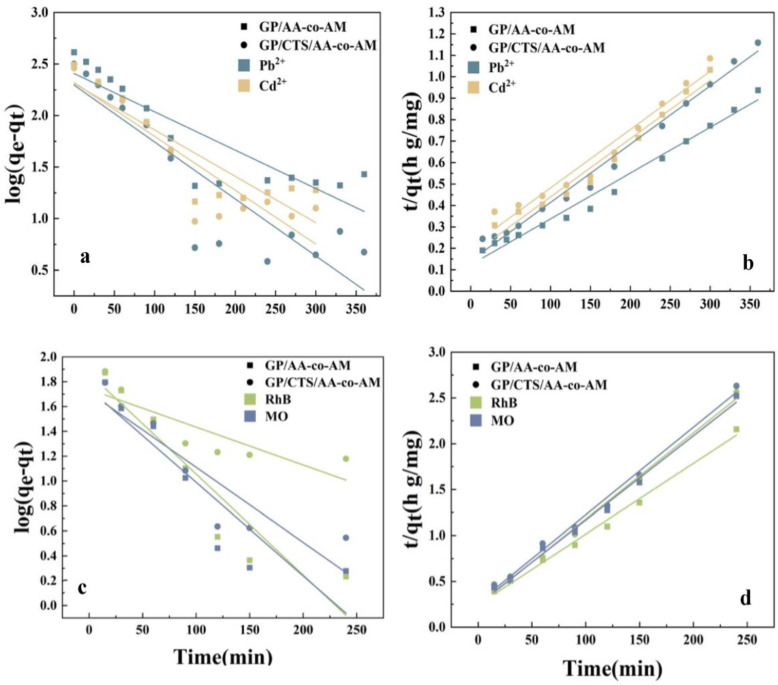
The pseudo-first-order (**a**,**c**) and pseudo-second-order (**b**,**d**) kinetic models of GP/AA-co-AM.

Figure 6 presents a meticulously fitted depiction of the two models. The kinetic parameters derived from the meticulous fitting of the empirical data to both kinetic models for the adsorption of the four pollutants are meticulously shown in Table S2. By conducting a judicious comparative analysis of the correlation coefficients (*R^2^*) governing the fitted curves for each distinct hydrogel and pollutant, it becomes manifestly apparent that the pseudo-second-order model provides a more precise exposition of the adsorption mechanisms for GP/AA-co-AM and GP/CTS/AA-co-AM, particularly concerning individual pollutants Pb^2+^, Cd^2+^, RhB, and MO. This observation underscored that the adsorption of individual pollutants onto the hydrogel surface entailed a series of intricate steps, encompassing diffusion towards the active sites nestled within the hydrogel and culminating in attaining adsorption equilibrium. It is suggested that the adsorption of heavy metal ions and dyes onto a hydrogel is fundamentally attributed to chemical adsorption.

As delineated by the intraparticle diffusion models presented in Figures S2 and S3 [50], as well as the comprehensive details tabulated in Tables S3 and S4, the adsorption mechanisms exhibited by GP/AA-co-AM and GP/CTS/AA-co-AM in relation to heavy metals and dyes manifest into discernible dual stages. In the initial phase, metal ions and dyes were diffused onto the adsorbent surface. The absence of line segment intersection indicated that the adsorption was influenced by both intra-particle and surface diffusion dynamics. In the subsequent stage, intra-particle diffusion became the pivotal factor for adsorption until equilibrium.

#### 2.3.3. Effect of Ion Concentration on Adsorption and Isothermal Models

**Figure 7:**
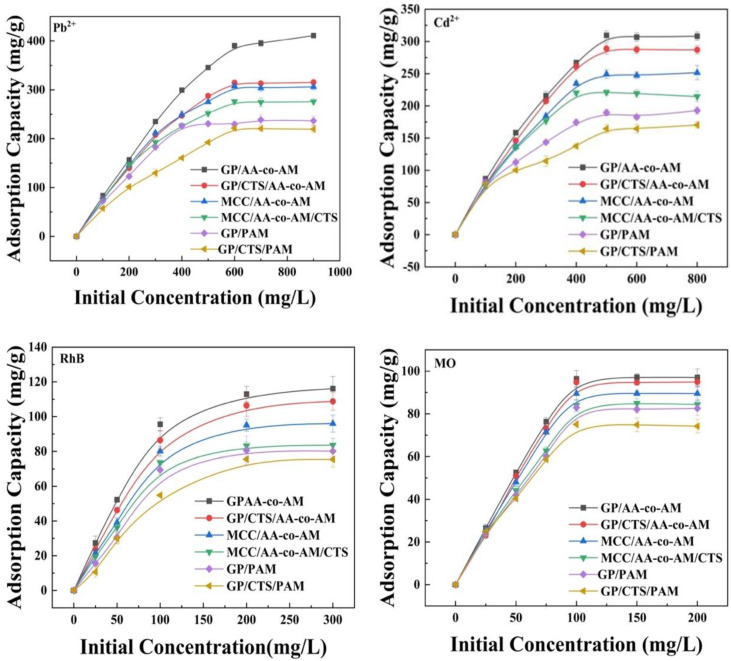
Effect of initial concentration on Pb^2+^, Cu^2+^, RhB, and MO adsorption. (Adsorption conditions: temperature, 25 °C; adsorbent, 20 mg; volume of adsorption solution, 50 mL; pH, 4.0 for Pb^2+^ and Cd^2+^, 3.0 for RhB and 8.0 for MO; contact time, 150 min.).

An extensive examination of the influence of the initial concentrations of Pb^2+^ and Cd^2+^ on the adsorption dynamics was meticulously undertaken across concentrations ranging from 100 to 1000 mg/L, as presented in Figure 7. The adsorption capacities exhibited an ascending trajectory for the prepared hydrogels as the initial concentrations were elevated. Equilibrium in the adsorption processes was realized upon the attainment of initial concentrations of 600 mg/L for Pb^2+^ and 500 mg/L for Cd^2+^. From this, it can be inferred that the binding sites in the hydrogels were almost entirely filled. At the optimized initial concentrations of 600 mg/L and 500 mg/L, the most substantial adsorption capacities were witnessed for GP/AA-co-AM and GP/CTS/AA-co-AM, precisely 411.1 mg/g and 314.6 mg/g for Pb^2+^ and 309.5 mg/g and 289.0 mg/g for Cd^2+^, respectively. In contrast, GP/PAM and GP/CTS/PAM displayed the least potent adsorption capacities among the hydrogel adsorbents, registering values of 238.6 mg/g and 221.7 mg/g for Pb^2+^ and 192.8 mg/g and 170.3 mg/g for Cd^2+^, respectively, under the optimal initial concentrations of 600 mg/L and 500 mg/L. This variance can be elucidated by the interactions forged between the active sites and metal ions at escalated initial concentrations. The augmented diffusion of metal ions into the adsorbent, propelled by the amplified concentration gradient, contributed to this phenomenon. Integrating -COOH groups derived from AA into GP/AA-co-AM, GP/CTS/AA-co-AM, MCC/AA-co-AM, and MCC/AA-co-AM/CTS further accentuated the propensity for heavy metal adsorption. This culminated in the comparatively diminished adsorption capacities observed for GP/PAM and GP/CTS/PAM upon exposure to the two heavy metals.

**Figure 8:**
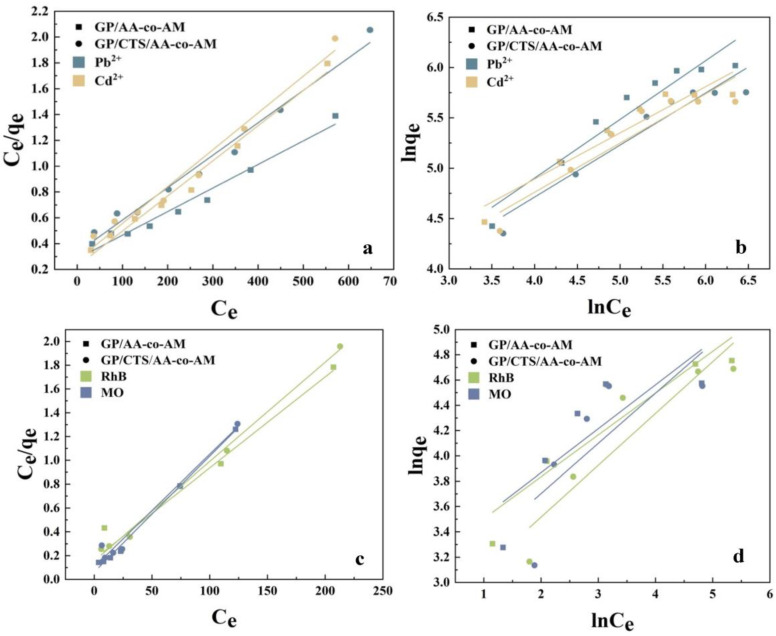
Langmuir (**a**,**c**) and Freundlich (**b**,**d**) isotherm models of heavy metal ion and dye adsorption on GP/AA-co-AM and GP/CTS/AA-co-AM.

The impact of varying the initial dye concentrations within a range of 25–300 mg/L on the adsorption phenomenon is effectively illustrated in Figure 8. Evidently, the adsorption quantity of the prepared hydrogel adsorbent exhibited an augmentation alongside the escalation in the initial dye concentrations. Specifically, for the hydrogels GP/AA-co-AM and GP/CTS/AA-co-AM, equilibrium was attained with a maximum adsorption capacity of 116.1 mg/g and 108.75 mg/g for RhB at an initial 200 mg/L concentration. Correspondingly, for MO, equilibrium was achieved with a maximal adsorption capacity of 97.16 mg/g and 94.94 mg/g, respectively, when exposed to an initial 100 mg/L concentration.

Adsorption isotherms are crucial for characterizing interfacial adsorption, which arises from the interaction between metal ions and adsorbent material surfaces. In this study, the analysis of the adsorption processes pertaining to GP/AA-co-AM and GP/CTS/AA-co-AM was facilitated through the application of the Langmuir and Freundlich isotherm models [51,52], respectively. The Langmuir model, designed for monolayer adsorption systems, revealed the presence of a constrained number of discrete adsorption sites. Conversely, the Freundlich model, accommodating the heterogeneous nature of the adsorption surface, proved more adept at characterizing systems involving multi-layer adsorption or those featuring elevated adsorption concentrations. The detailed equations associated with the Langmuir and Freundlich isotherm models and the thermodynamic study results are exhibited in the Supplementary Information. 

Figure 8, along with the comprehensive details provided in Table S5, offers a comprehensive exposition of the fitting parameters and associated curves corresponding to both models. Guided by the correlation coefficient (*R*^2^), the Langmuir model emerged as the more optimal fit, distinctly indicating the prevalence of monolayer adsorption as the dominant mechanism governing the adsorption process. Examining Table S6 reveals that all Δ*G*◦ values are negative, indicating that the adsorption processes of GP/CTS/AA-co-AM for Pb^2+^ and RhB are entirely spontaneous. The positive ΔS◦ values suggest an increase in the system entropy following adsorption [53]. Furthermore, the positive ΔH◦ value denotes the endothermic nature of the process.

### 2.4. Selective Adsorption of Pollutants

**Figure 9:**
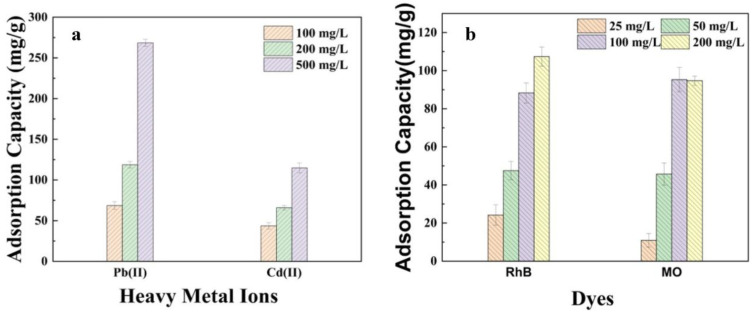
The competitive adsorption of GP/CTS/AA-co-AM for (**a**) Pb(II) and Cd(II) and (**b**) RhB and MO in their mixture solution.

To explore the selective adsorption tendencies of the hydrogels, an in-depth analysis of the adsorption performance of GP/CTS/AA-co-AM was conducted in mixtures encompassing both heavy metals and dyes, as elucidated in Figure 9. The hydrogel exhibited distinctive selective adsorption characteristics, specifically concerning heavy metals(Figure 9a). GP/CTS/AA-co-AM showcased a higher adsorption capacity for Pb^2+^ when mixed with Cd^2+^ across varying mixture concentrations. At a mixture concentration of 500 mg/L, the adsorption capacity of GP/CTS/AA-co-AM for Pb^2+^ (268.4 mg/g) notably surpassed that for Cd^2+^ (114.8 mg/g). Nonetheless, within the mixture, the adsorption capacity of the hydrogel for both Pb^2+^ and Cd^2+^ was comparatively lower than the adsorption capacity observed when each metal ion was subjected to individual testing. This selective adsorption propensity towards Pb^2+^ can be ascribed to the higher affinity exhibited by Pb^2+^ ions for active functional groups, such as -COOH, NH_2_, and -OH, in comparison to Cd^2+^ ions. Additionally, these functional groups contribute to the hydrophilicity and surface polarity of the hydrogels [39]. Furthermore, this preference can also be attributed to the larger ionic radius of Pb^2+^ (0.118 nm) as opposed to Cd^2+^ (0.097 nm), along with the smaller hydrated radius (Pb^2+^ = 0.401 nm, Cd^2+^ = 0.426 nm) and hydration energy (Pb^2+^ = −1481 kJ/mol, Cd^2+^ = −1807 kJ/mol) associated with Pb^2+^ [54]. As a result, Pb^2+^ ions exhibit a heightened affinity and a greater propensity for adsorption when compared to Cd^2+^ ions. The hydrogel displayed substantial adsorption capacities for the RhB and MO dyes, reaching 107.4 mg/g and 94.7 mg/g, respectively, at a 200 mg/L mixture concentration (Figure 9b).

Unlike the behavior observed with heavy metals, the presence of a dye mixture did not influence the individual adsorption performance of each dye. These findings suggested that GP/CTS/AA-co-AM was a promising adsorbent for selectively removing heavy metal ions and maintaining stable adsorption performance for dyes in the presence of competing species. The observed selective adsorption behavior, driven by distinct affinities and interactions, highlighted the potential applications of this type of hydrogel in environmental remediation and wastewater treatment scenarios. The intricate impact of ionic strength on the adsorption capacity of the adsorbents is delineated in Figure S3 of the Supplementary Information.

### 2.5. Regeneration of GP/CTS/AA-co-AM

**Figure 10:**
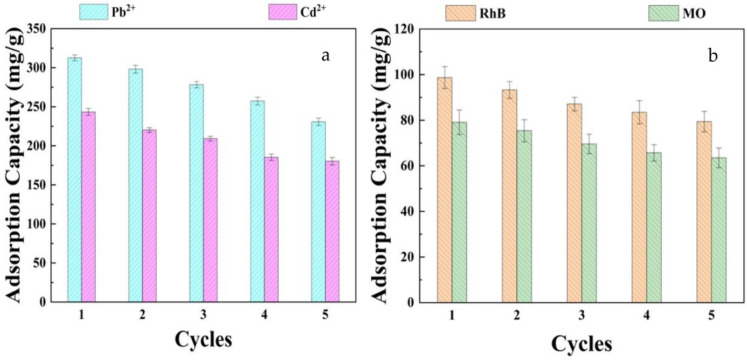
Regeneration of GP/CTS/AA-co-AM for (**a**) Pb^2+^ and Cd^2+^ and (**b**) RhB and MO.

The assessment of hydrogel regenerative efficacy holds significant importance in gauging the practical viability of adsorbents. This investigation meticulously scrutinized the regenerative potential of GP/CTS/AA-co-AM concerning the four pollutants across five consecutive adsorption–desorption cycles. In the inaugural cycle, the measured adsorption capacities were established as the baseline and assigned a value of 100%. After this, the adsorption capacity ratio for each cycle was calculated in relation to the capacity of the baseline cycle. This computation was a means to evaluate the aptitude of the hydrogel for regeneration. As depicted in Figure 10, there is a progressive decline in the adsorption capacities across successive cycles. Nonetheless, even after undergoing five cycles of sequential adsorption and desorption, the adsorption capacities remain notably above 70% for Pb^2+^ and Cu^2+^ ions (Figure 10a) and beyond 80% for RhB and MO (Figure 10b). This observation cogently suggested that the hydrogel founded on GP-based material enables facile recovery and showcases commendable regenerative capabilities. These results show that GP/CTS/AA-co-AM is a useful, effective, and reusable adsorbent specifically designed for removing pollutants. This material has great potential for use in sustainable wastewater treatment and environmental remediation projects.

### 2.6. Proposed Mechanism of Adsorption for Dyes

**Figure 11:**
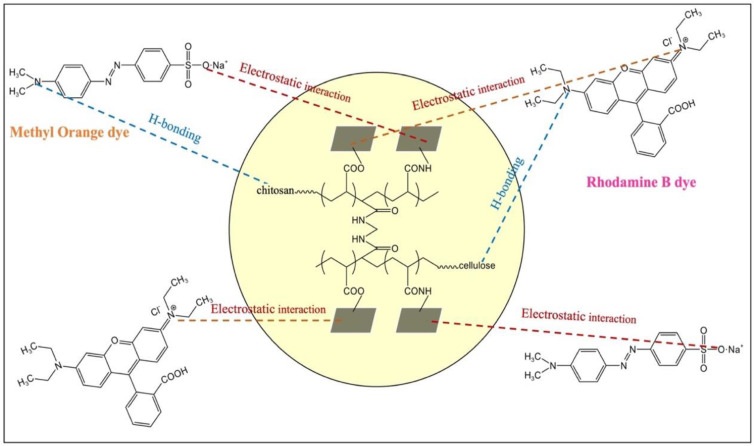
The proposed mechanism of adsorption for RhB and MO.

The intricacies of heavy metal adsorption mechanisms have been exhaustively addressed in our prior research [28]. As a result, the current investigation focuses exclusively on comprehending the interaction governing the adsorption of RhB and MO dyes onto the GP/CTS/AA-co-AM hydrogel (Figure 11). RhB, distinguished by negatively charged carboxylate groups (COO^−^) at lower pHs, evinces an inclination towards electrostatic interactions with the hydrogel due to the presence of cationic functional groups on its surface. The elevation of pH> 4 leads to the presence of COO^–^ groups on the hydrogel surface, culminating in a reduced adsorption propensity for RhB, largely due to the repulsive forces arising from anion–anion interactions. Conversely, MO, as an anionic dye, displays a diminished likelihood of interaction with the hydrogel on account of the presence of anionic functional groups on its surface. Upon completion of the dye adsorption process, the peaks within the spectral region spanning 1380–1460 cm^−1^ become conspicuous by their absence in the FTIR spectrum of the hydrogel. This observation distinctly signifies that a discernible interaction has transpired between the hydrogel and the dyes, resulting in the obliteration of characteristic peaks.

When RhB and MO establish contact with a hydrogel with a negative surface charge (Figure S4, Supplementary Information), the -COOH and -NH_2_ functional groups serve as recipients for hydrogen bonding. These electronegative constituents house lone pairs of electrons, generating a partial negative charge that orchestrates the attraction of positively charged hydrogen atoms within the dye molecules, facilitating the formation of hydrogen bonds between the hydrogel and the dye molecules. Overall, the GP/CTS/AA-co-AM hydrogel primarily engages in the adsorption of RhB and MO molecules through electrostatically alluring interactions and the establishment of hydrogen bonding networks.

Table 1 presents an extensive and comparative evaluation of the hydrogels synthesized within the ambit of this study, compared with other adsorbents in the published literature. The findings underscore the distinctly elevated adsorption capacities exhibited by the hydrogels synthesized in this study, specifically in relation to the four designated pollutants—Pb^2+^, Cd^2+^, RhB, and MO. This salient observation accentuates the considerable potential inherent within the GP-based hydrogel (GP/CTS/AA-co-AM) that was meticulously developed within this research endeavor. This accomplishment positions the hydrogel as an exceptionally promising adsorbent, offering compelling efficacy in efficiently sequestering heavy metals and dyes from aqueous solutions. The adsorption capacity and rate make it ideal for addressing water pollution challenges. Its attributes make it suitable for tackling multifaceted water quality degradation issues.

## 3. Conclusions

Cellulose-based hydrogels presented a promising avenue for mitigating water pollution. The synthesized GP/CTS/AA-co-AM hydrogel exhibited efficiency in the removal of Pb^2+^, Cd^2+^, RhB, and MO from aqueous solutions. With its enhanced mechanical properties, the hydrogel demonstrated excellent performance in adsorbing these pollutants, achieving maximum adsorption capacities under specific operating conditions. At a pH of 4.0, a contact time of 120 min, and initial concentrations of 600 mg/L for Pb^2+^ and 500 mg/L for Cd^2+^, the maximum adsorption capacities reached 314.6 mg/g and 289.1 mg/g, respectively. Additionally, for the dyes, the GP/CTS/AA-co-AM hydrogel exhibited adsorption capacities of 106.4 mg/g for RhB and 94.8 mg/g for MO, consistently maintaining a stable adsorption capacity across various pH levels. The adsorption processes of the hydrogels for the pollutants were described by pseudo-second-order kinetics and the Langmuir model, validating the appropriateness of the synthesized hydrogels for water remediation purposes. Moreover, the hydrogel exhibited selective adsorption for Pb^2+^ and an excellent retention of adsorption capacities (above 70% for heavy metals and above 80% for dyes) even after undergoing five consecutive regeneration cycles, showing its reusability and long-term economic viability. Overall, this research demonstrated the significant potential of the GP/CTS/AA-co-AM hydrogel as a viable and efficient solution for removing heavy metals and organic dyes from water sources. The study paves the way for sustainable and eco-friendly water purification strategies, contributing to the protection of both human health and the ecosystem.

## 4. Materials and Methods

### 4.1. Materials

Highly viscous chitosan (CTS) extracted from shrimp shells was sourced from Shanghai Macklin Biochemical Co., Ltd. (M = 1526.46 g/mol, 100–200 mPa.s, deacetylation degree >75%, Shanghai, China). GP prescription was obtained from Huiren Tang Pharmacy (Lanzhou, China). Microcrystalline cellulose (MCC), sodium hydroxide (NaOH), acrylamide (AM), acrylic acid (AA), N, N-methylene bisacrylamide (MBA), Rhodamine B (RhB), and methyl orange (MO) were procured from Tianjin Damao Chemical Reagent Factory (Tianjin, China). Urea (CH_4_N_2_O) was sourced from Yantai Shuangshuang Chemical Co., Ltd, (Yantai, China). Absolute ethanol (C_2_H_6_O) and ammonium persulfate (APS) were acquired from Tianjin Best Chemical Co., Ltd. (Tianjin, China). Lead nitrate (Pb (NO_3_)_2_) and cadmium chloride (CdCl_2_) were obtained from Tianjin Kermel Chemical Reagent Co., Ltd. (Tianjin, China). All other chemical compounds employed in this study were of analytical grade and were utilized without the requirement for additional purification.

### 4.2. Synthesis of Hydrogels

#### 4.2.1. Pretreatment of GP

The GP material underwent pretreatment according to the methodology reported in our previous works [22]. The GP sample was initially rinsed with distilled water and subjected to three successive boiling cycles to eliminate the active constituents. The dried GP was subjected to a modification process employing a NaOH solution (1 mol/L) for 8 h at 40 °C. After drying and grinding, the resulting GP-NaOH powder was sieved through a 200-mesh screen called GP-NaOH. 

#### 4.2.2. Dissolution of GP/CTS and MCC

A mixture comprising 5 g of GP-NaOH and varying quantities of CTS, ranging from 0.5 g to 2.5 g with increments of 0.5 g, was homogeneously dispersed within an alkaline/urea solution at room temperature. Subsequently, the mixture underwent magnetic agitation at a speed of 200 rpm for 2 h. Following this, the mixtures were subjected to a freezing process at −20 °C for a period of 8 h, followed by a thawing step combined with stirring at 0 °C for 2 h. This cycle of freezing and thawing was iterated twice. The resulting opaque solution underwent microwave irradiation for 20 min and was then centrifuged at 4000 rpm for 5 min. The supernatants obtained were decanted, and the pH was adjusted to a neutral level using a 1 M HCl solution. By employing the same procedure, solutions containing solely GP and combinations of GP with CTS (GT/CTS-0.5, GT/CTS-1, GT/CTS-1.5, GT/CTS-2, GT/CTS-2.5) were prepared and subsequently stored at a temperature of 4 °C.

To serve as a control experiment in the production of hydrogels based on GP, hydrogels were also prepared utilizing MCC. To dissolve the MCC, 5 g of MCC was dispersed in an alkaline/urea solution in an ambient environment. The mixture underwent magnetic stirring at a speed of 200 rpm for 2 h, followed by a freezing cycle at −20 °C for 8 h, and thawing with stirring at 40 °C for an additional 2 h. This freezing and thawing process was repeated twice. Subsequently, the resulting mixture was exposed to microwave irradiation for 20 min. Then, 1 M of HCl solution was added to adjust the pH to near neutral. The resulting mixtures were stored at a temperature of 4 °C.

#### 4.2.3. Hydrogel Synthesis

**Figure 12:**
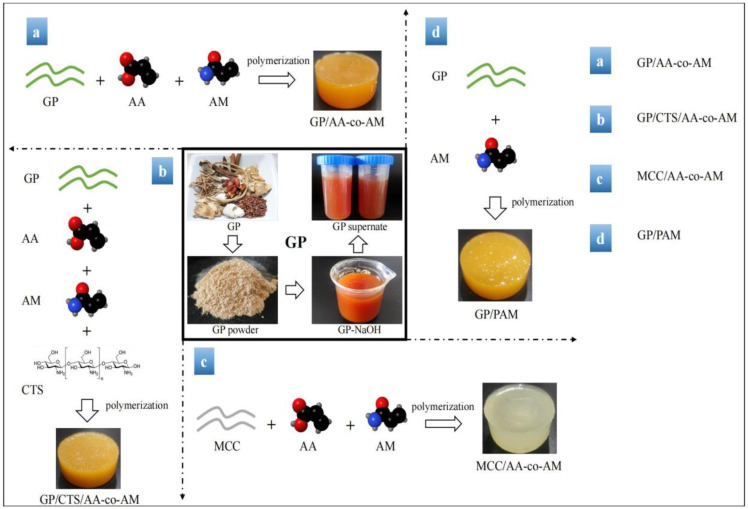
The preparation process of hydrogels.

From our preliminary experimental results, the hydrogels produced through the amalgamation of GP/CTS-2 and GP/CTS-2.5 showcased remarkable mechanical attributes and adsorption efficiency. Subsequent iterations were guided by a resource-efficient approach based on our previous study [28], employing 2 g of CTS in the synthesis of the ensuing hydrogels. It is noteworthy to mention that in the subsequent study, the reference to GP/CTS specifically pertained to GP/CTS-2. The GP/CTS solution was subjected to stirring for 15 min at room temperature in the presence of AM (2 g), AA (3 g), and MBA (0.2 g). Subsequently, an appropriate volume of APS solution was added, and the resulting mixture was stirred for 1 min before allowing 20 min of gelation at room temperature. Following this reaction, samples were cleansed with absolute ethanol and distilled water to remove residual reagents and by-products, and then they were dried at 60 °C for 8 h. The synthesis procedures and chemical compositions for the various hydrogels are elaborated in Figure 12 and Table S1.

### 4.3. Characterization

The compositional analysis of the hydrogels was carried out using Fourier-transform infrared spectroscopy (FTIR, Nicolet Nexus, Waltham, MA, USA) over a spectral range of 4000 cm^−1^ to 400 cm^−1^. The surface characteristics and structural configuration of the desiccated hydrogel covered with gold film were investigated via scanning electron microscopy (SEM; JSM-6701F, JEOL, Tokyo, Japan). The thermal stability assessment of the powdered specimens was performed using thermogravimetric analysis (TGA) with a PerkinElmer TGA-7 thermogravimetric instrument (PerkinElmer Cetus Instruments, Nor-walk, CT, USA). The crystalline arrangement of the samples was ascertained by employing X-ray diffraction (XRD) with a D8-Advance instrument (Bruker, Saarbrücken, Germany). The mechanical properties of the hydrogels were tested through the preparation of 10 mm dumbbell-shaped specimens with an inner width of 5 ± 0.15 mm, and the ultimate elongation at rupture (%) was determined using a universal testing machine (INSTRON model 1405) operating at a deformation rate of 5 mm/min.

### 4.4. Swelling Ratio at Different pHs

To explore the hydrogel swelling response across varying pH conditions, 10 mg of dried hydrogel samples were immersed in 50 mL solutions spanning from pH 1 to 9 at room temperature, modulated with 0.1 M of HCl and NaOH. Post-immersion, the weights of the swollen hydrogels were quantified. The swelling ratio (*S_W_* g/g) of the hydrogel was ascertained utilizing Equation (1):

(1) $$S_{w}=(W_{s}-W_{d})/W_{d}$$

where *S_W_* (g/g) represents the swelling ratio of the hydrogel, *W_d_* (g) is the weight of the dried hydrogel, and *W_S_* (g) is the weight of the swollen hydrogel.

### 4.5. Adsorption Performance

The adsorption experiments aimed to assess the adsorption capacities of a range of hydrogel adsorbents for the purpose of pollutant removal. The experimental procedure entailed introducing 0.02 g of hydrogel samples into colorimetric tubes with 25 mL of aqueous solutions containing individual pollutants (Pb(NO_3_)_2_, Cd(Cl)_2_, RhB, and MO) or combinations thereof. The tubes were sealed carefully and agitated at 120 rpm for 2 h (25 °C) to achieve optimal reaction conditions. To investigate the effect of pH on pollutant adsorption, solutions were adjusted to a pH range of 1.0–8.0 using 0.1 M of HCl or 0.1 M of NaOH.

The residual concentrations of the pollutants in the solutions were measured using atomic absorption spectrometry (AAS, PerkinElmer PinAAcle 900 T, USA) for Pb^2+^ and Cd^2+^ and UV–visible spectrophotometry (UV1900, Shimadzu, Japan) for RhB (λ_max_ 554 nm) and MO (λ_max_ 430 nm). The calculation of the equilibrium adsorption capacity (*q_e_*, mg/g) and the amount of pollutants adsorbed at a given time (*q_t_*, mg/g) was achieved by employing the equations provided in the Supplementary Information.

The experiment measured the adsorption kinetics of hydrogel–pollutant mixtures for 15 to 350 min. The dataset for kinetic adsorption was used to fit both pseudo-first-order and second-order models, which provided insights into the adsorption process. For the adsorption isotherm experiments(Figure S5, Supplementary Information), the concentration spectrum of metal ions encompassed values ranging from 100 to 900 mg/L, while for the dye solutions, the concentration scale extended from 25 to 300 mg/L. To determine the adsorption characteristics, Langmuir and Freundlich isotherms were used to analyze equilibrium experimental data from various initial concentrations of metal ions and dye solutions.

In the desorption experiment, hydrogel adsorbents loaded with four pollutants (Pb^2+^, Cd^2+^, RhB, and MO) were mixed with 0.5 M of HCl solution and agitated on an orbital shaker for 60 min, followed by washing and vacuum drying at 40 °C. Adsorbents were regenerated through five consecutive adsorption–desorption cycles. Each experiment was conducted in triplicate, and the outcomes presented herein are indicative of the mean values obtained.

### 4.6. Selective Adsorption Experiments in Binary Systems

A solution containing a mixture of Pb^2+^ and Cd^2+^ ions was prepared by introducing 0.02 g of GP/CTS/AA-co-AM into a 25 mL volume. The initial concentrations of Pb^2+^ and Cd^2+^ were kept at 100 mg/L, 200 mg/L, and 500 mg/L, respectively. Additionally, the selective adsorption of GP/CTS/AA-co-AM hydrogels in a mixture of RhB and MO was investigated. The initial concentrations of RhB and MO were set at 25 mg/L, 50 mg/L, 100 mg/L, and 200 mg/L, respectively.

## Figures and Tables

**Table 1:** Comparison of adsorption of heavy metal ions by different adsorbents.

Adsorbents	*q_e_* (mg/g)				References
	Pb^2+^	Cd^2+^	RhB	Mo	
GP/CTS/AA-co-AM	314.58	289.10	106.39	94.77	This work
Manganese dioxide-poly(N-hydroxymethyl acrylamide/2-hydroxyethyl acrylate)	100.28	52.61	-	-	[55]
Acrylamide/acrylic acid cellulose	393.28	289.97	-	-	[40]
Carboxymethyl cellulose/polyacrylamide	312.50	256.41	-	-	[56]
Carboxylated cellulose nanofibrils-filled magnetic chitosan	171.00	-	-	-	[57]
Chitosan/poly(ethylene glycol)/ acrylamide	-	-	-	185.24	[58]
Thiourea-grafted-chitosan	-	134.00	-	-	[59]
Poly(2-(2-methoxyethoxy) ethyl methacrylate-co-oligo (ethylene glycol) methacrylate-co-acrylic acid) with attapulgite/Fe_3_O_4_	-	-	1.65	-	[60]
Furfural residue	-	-	37.93	54.95	[61]
2-hydroxyterephthalic acid/hypercross-linked polymer	178.27	69.69	133.46	423.22	[62]
Poly-dopamine/graphene oxide	53.60	33.30	-	-	[63]
Lansium domesticum Shell	-	-	11.58	3.84	[64]
Tri-metallic layered double hydroxides	-	-	38.03	32.67	[65]

## Data Availability

All data and materials are available on request from the corresponding author. The data are not publicly available due to ongoing research using a part of the data.

## Supplementary Materials

The following supporting information can be downloaded at https://www.mdpi.com/article/10.3390/gels10020142/s1: Table S1. Chemical formulas of synthesized hydrogels and the compressive mechanical properties of hydrogels (σ and E are strains at break and Young’s modulus under the compression model, respectively). Table S2. Kinetic parameters for the adsorption of heavy metal ions. Table S3. The parameters of intraparticle diffusion of GP/AA-co-AM and GP/CTS/AA-co-AM for RhB and MO. Table S4. The parameters of intraparticle diffusion of GP/AA-co-AM and GP/CTS/AA-co-AM for Pb^2+^ and Cd^2+^. Table S5. Adsorption isotherms of heavy metal ions on hydrogel. Table S6. Thermodynamic parameters of LR-EPI-8 for Pb (II) and Cu (II). Figure S1. SEM of GP/AA-co-AM before (a), after the adsorption for MO (b) and Pb^2+^(c); and GP/CTS/AA-co-AM before (d) and after the adsorption for MO (e,f); and GP/CTS/AA-co-AM before (g) and after the adsorption for Pb^2+^ (e,f). Figure S2. The intraparticle diffusion of GP/AA-co-AM and GP/CTS/AA-co-AM for RhB and MO. Figure S3. The effect of ion strength on the adsorption capacities of GP/CTS/AA-co-AM for Pb^2+^ and Cd^2+^. Figure S4. The zeta potential of GP/CTS/AA-co-AM at different pH. Figure S5. Adsorption thermodynamic for adsorption of Pb2+(a) and RhB (b) on GP/CTS/AA-co-AM.
