# Supplementary material for: Efficient and Selective Removal of Heavy Metals and Dyes from Aqueous Solutions Using Guipi Residue-Based Hydrogel
Source: Gels. 2024 Feb 13;10(2):142. doi: 10.3390/gels10020142 (PMC10887816; doi:10.3390/gels10020142)
Supplement: Supplementary file 1 [file gels-10-00142-s001.zip › gels-2839870-supplementary.pdf]

Efficient and Selective Removal of Heavy Metals and Dyes from Aqueous Solutions Using Guipi Residue-Based Hydrogel

Xiaochun Yin *, Pei Xu and Huiyao Wang *

Department of Civil Engineering, New Mexico State University, Las Cruces, NM 88003, USA; pxu@nmsu.edu
* Correspondence: lzyxc@nmsu.edu (X.Y.); huiyao@nmsu.edu (H.W.)

1. The chemical formulas of synthesized hydrogels and their mechanical property

Table S1. Chemical formulas of synthesized hydrogels and the compressive mechanical properties of hydrogels (σ and *E* are strains at break and Young's modulus under the compression model, respectively).

Samples	Materials (g)	CTS (g)	AM (g)	AA (mL)	*E* [MPa]	*σ* [MPa]
GP/AA-co-AM	5	0	2	3	0.12	0.07
GP/CTS/AA-co-AM	5	2	2	3	0.25	0.21
MCC/AA-co-AM	5	0	2	3	0.11	0.07
MCC/AA-co-AM/CTS	5	2	2	3	0.18	0.14
GP/PAM	5	0	3	0	0.08	0.07
GP/CTS/PAM	5	2	3	0	0.16	0.11

2. The equations of calculating adsorption capacities

The adsorption capacity of the hydrogels for Pb^{2+}, Cd^{2+}, RhB, and MO at a specific time (q_t, mg/g) and equilibrium (q_e, mg/g) was determined by the following equations:

$$q_e = \frac{(C_0 - C_e)V}{W} \qquad (S1)$$

$$q_t = \frac{(C_0 - C_t)V}{W} \qquad (S2)$$

where, q_e (mg / g) is the equilibrium adsorption capacity; q_t (mg / g) is the adsorption capacity at a specific time; C_0 (mg/L) is the initial concentration of pollutants; C_e (mg/L) is the equilibrium concentration of pollutants; C_t (mg/L) is the concentration of pollutants at time t (h), V (mL) is the volume of adsorption solution, and W(g) is the initial mass of the dry sample W_t (mg).

3. The SEM of hydrogels before and after adsorption for Pb^{2+} and MO

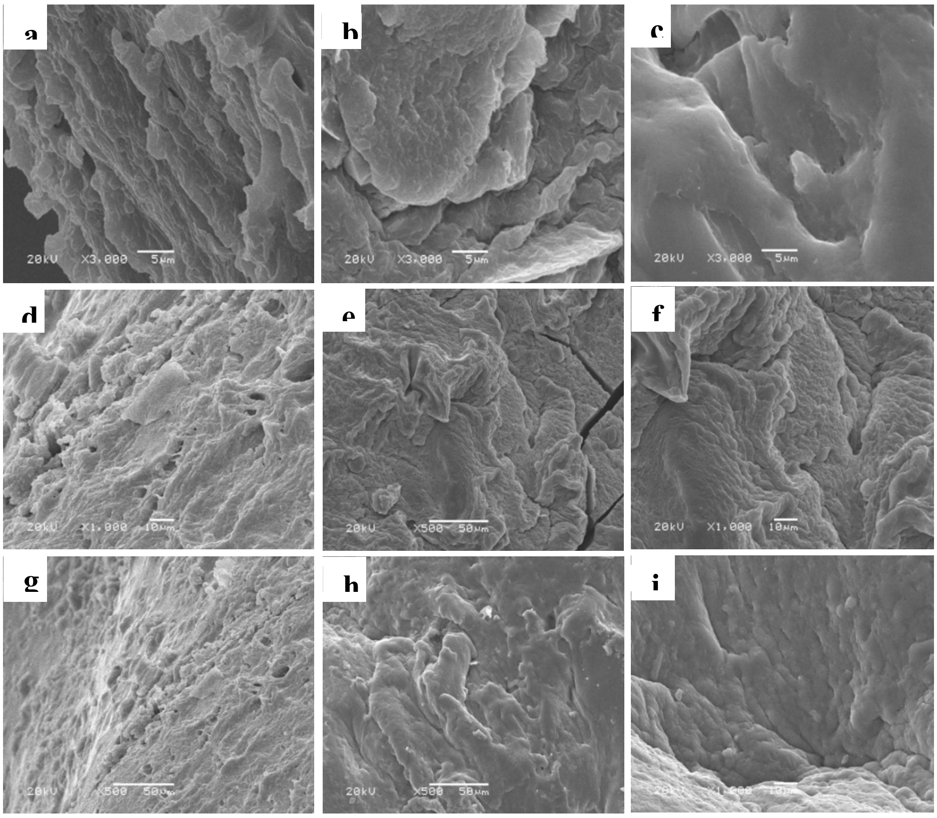

Figure S1. SEM of GP/AA-co-AM before (a), after the adsorption for MO (b) and Pb^{2+}(c); and GP/CTS/AA-co-AM before (d) and after the adsorption for MO (e, f); and GP/CTS/AA-co-AM before (g) and after the adsorption for Pb^{2+} (e, f).

4. Adsorption Kinetics

The kinetic adsorption data were fitted by pseudo-first order and pseudo-second order kinetic models, respectively, which are represented by Eq.(S3) and (S4):

$$\log(q_e - q_t) = logq_e - \frac{k_1}{2.303}t \qquad (S3)$$

$$\frac{t}{q_t} = \frac{1}{k_2 {q_e}^2} + \frac{t}{q_e} \qquad (S4)$$

where q_e (mg/g) is the adsorption capacity at equilibrium, q_t (mg / g) is the adsorption capacity at specific time, and k_1 (min $^{-1}$) and k_2 (g / mg -min)) are the quasi-first and pseudo-secondary rate constants, respectively.

Table S2. Kinetic parameters for the adsorption of heavy metal ions.

	**pseudo-first-order**			pseudo-second-order		
	k_1(min^{-1})	***q_{e1}(mg/g)***	***R^2***	k_2(g/mg-min)	*q_{e2}(mg/g)*	*R^2*
GP/AA-co-AM(Pb^{2+})	0.0080	255.82	0.7671	0.3×10^{-4}	467.29	0.9826
GP//CTS AA-co-AM (Pb^{2+})	0.0130	197.51	0.7921	0.3×10^{-4}	367.65	0.9865
GP/AA-co-AM(Cd^{2+})	0.0100	204.04	0.7601	0.4×10^{-4}	367.65	0.9664
GP/CTS/AA-co-AM (Cd^{2+})	0.0120	209.35	0.7779	0.1×10^{-4}	367.65	0.9548
GP/AA-co-AM(RhB)	0.0176	64.60	0.7672	0.3×10^{-3}	126.26	0.9912
GP/CTS/AA-co-AM(RhB)	0.0067	52.97	0.5824	0.4×10^{-3}	104.49	0.9871
GP/AA-co-AM(MO)	0.0159	47.96	0.6448	0.3×10^{-3}	107.75	0.9898
GP/CTS/AA-co-AM(MO)	0.0139	51.70	0.7798	0.3×10^{-3}	104.71	0.9916

5. Intra-particle diffusion model

The intra-particle diffusion model is useful in studying the diffusion process that happens in the surface layer during adsorption kinetics. By using this model, we can better understand how substances diffuse in the surface layer and how it affects other materials. We have extensively researched this model to gain insights into the diffusion mechanism during adsorption. This model considers the availability of adsorption sites and mass transfer processes, which are the main factors that affect adsorption kinetics. The equation for the intra-particle diffusion model is as follows (1) .

$$Q_t = k_i t^{1/2} + C \qquad \text{(S5)}$$

where, Qt (mg/g) is the adsorption capacity at a specific time; ki (mg/g·min$^{0.5}$) is the intra-particle diffusion constant associated with the absorption rate; and C (mg/g) is the intercept.

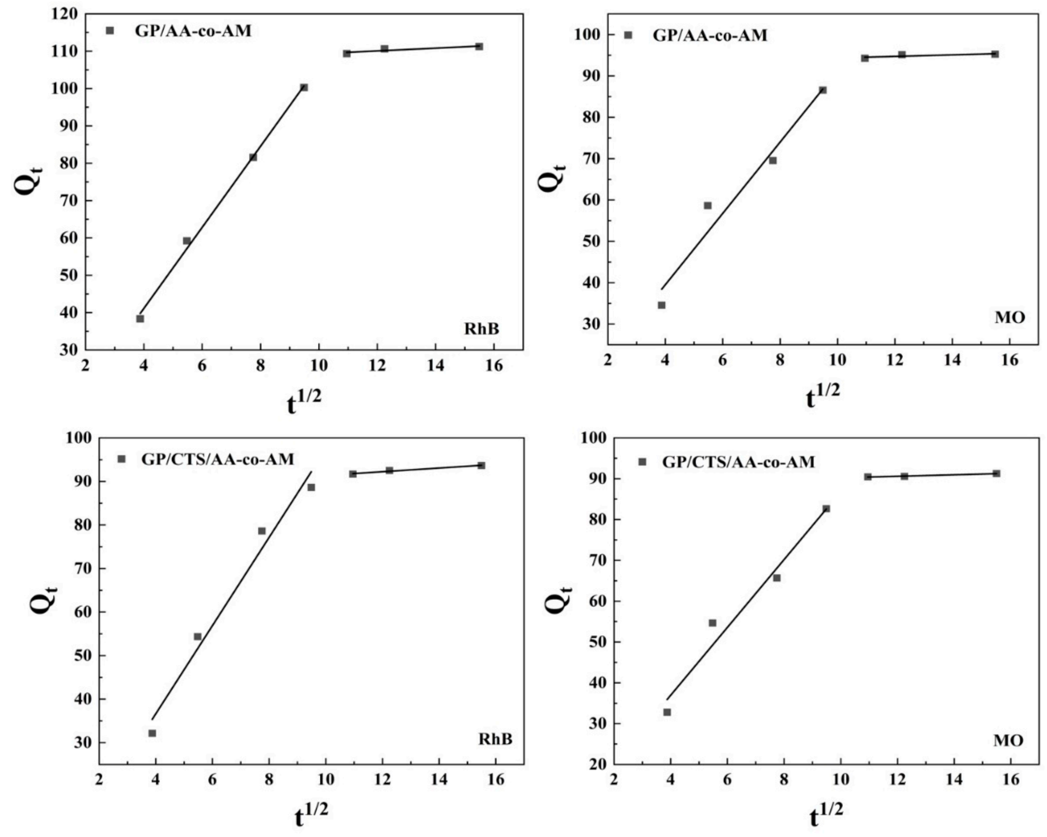

Figure S2. The intraparticle diffusion of GP/AA-co-AM and GP/CTS/AA-co-AM for RhB and MO.

Table S3. The parameters of intraparticle diffusion of GP/AA-co-AM and GP/CTS/AA-co-AM for RhB and MO

	k_{i1} (mg/g·min$^{0.5}$)	C_1 *(mg/g)*	R^2	k_{i2} (mg/g·min$^{0.5}$)	C_2 *(mg/g)*	R^2
GP/AA-co-AM(RhB)	10.86	-2.30	0.9955	0.37	105.65	0.6349
GP/AA-co-AM(MO)	8.62	5.04	0.9357	0.18	92.50	0.2542
GP/CTS/AA-co-AM (RhB)	10.13	-3.85	0.9634	0.42	87.18	0.9547
GP/CTS/AA-co-AM (MO)	8.31	3.74	0.9485	0.18	88.39	0.9535

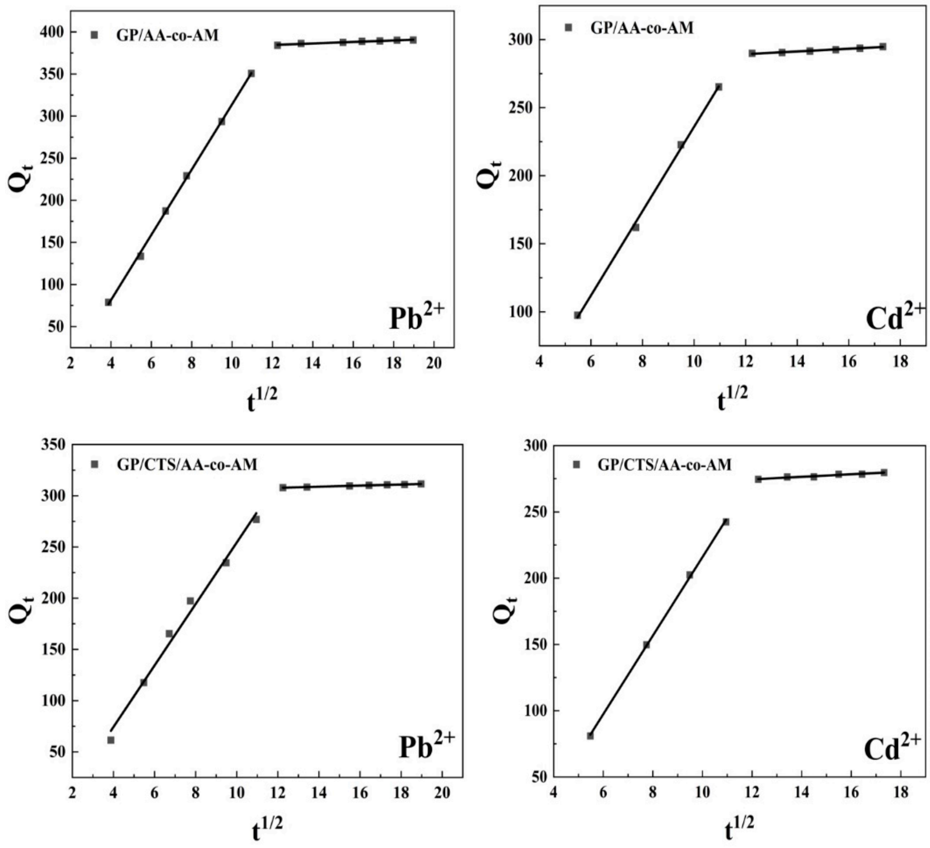

Figure S3. The intraparticle diffusion of GP/AA-co-AM and GP/CTS/AA-co-AM for Pb^{2+} and Cd^{2+}.

Table S4. The parameters of intraparticle diffusion of GP/AA-co-AM and GP/CTS/AA-co-AM for Pb^{2+} and Cd^{2+}.

	k_{i1} (mg/g·min$^{0.5}$)	C_1 *(mg/g)*	R^2	k_{i2} (mg/g·min$^{0.5}$)	C_2 *(mg/g)*	R^2
GP/AA-co-AM(Pb^{2+})	38.76	-73.62	0.9990	0.90	373.74	0.9685
GP/AA-co-AM(Cd^{2+})	30.98	-73.89	0.9975	0.99	277.39	0.9802
GP/CTS/AA-co-AM/ (Pb^{2+})	30.00	-45.71	0.9859	0.54	301.23	0.9973
GP/CTS/AA-co-AM/ (Cd^{2+})	29.62	-80.36	0.9992	0.96	263.00	0.9329

6. Adsorption Isotherm

The Langemuel and Flendrich isotherms are represented by Eq. (S6) and (S7):

$$\frac{C_e}{q_e} = \frac{1}{K_L q_m} + \frac{C_e}{q_m} \qquad (S6)$$

$$ln\, q_e = ln\, K_F + \frac{1}{n} ln\, C_e \qquad (S7)$$

where, q_e (mg/g) is the equilibrium adsorption capacity, C_e (mg/L) is the equilibrium concentration, K_L is the Langmuir constant, q_m (mg/g) is the maximum adsorption capacity covering the whole surface, R_L is the separation coefficient or the equilibrium parameter, C_0 (mg/L) is the initial concentration of heavy metal ions, and K_F and n are the Freundlich constant.

Table S5. Adsorption isotherms of heavy metal ions on hydrogel.

	Langmuir				Freundlich		
	q_m(mg/g)	K_L(L/mg)	R_L	R^2	n	$K_F(mg^{1-1/n}\ L^{1/n}\ g^{-1})$	R^2
GP/AA-co-AM(Pb^{2+})	546.5	0.006	0.217	0.9703	1.710	13.070	0.9069
GP/CTS/AA-co-AM(Pb^{2+})	398.4	0.008	0.172	0.9777	1.940	14.120	0.8996
GP/AA-co-AM(Cd^{2+})	367.7	0.012	0.143	0.9844	2.180	21.310	0.8907
GP/CTS/AA-co-AM(Cd^{2+})	350.9	0.011	0.154	0.9727	2.040	16.570	0.8571
GP/AA-co-AM(RhB)	130.6	0.045	0.100	0.9688	3.021	23.876	0.8114
GP/CTS/AA-co-AM(RhB)	119.8	0.054	0.085	0.9965	2.440	14.825	0.8067
GP/AA-co-AM(MO)	102.9	0.155	0.061	0.9957	2.880	23.882	0.5941
GP/CTS/AA-co-AM(MO)	105.7	0.090	0.100	0.9772	2.518	18.309	0.4488

7. The effect of the ionic strength

Owing to the inherent properties of NaCl, which serves to moderate electrostatic interactions between the adsorbents (GP/CTS-AA-co-AM) and contaminants, the effect of Na^+ and Cl^- on the adsorption performance of the adsorbents was analyzed. Various concentrations of NaCl, ranging from 1g/L to 10g/L, were employed to evaluate the impact of ionic strength on the adsorption capacity of the adsorbents. As illustrated in Figure S3, the presence of NaCl resulted in a reduction of the adsorption capacities of the adsorbents for heavy metals. The reasons are (1) the electrostatic shielding effect exerted by Na^+ and Cl^- ions and (2) the competition of Na^+ ions for binding sites [1]. In the context of dye adsorption, the initial adsorption capacity of GP/CTS-AA-co-AM for RhB increased. However, when the NaCl concentration reached 5 g/L, the efficiency of adsorption decreased due to the electrostatic shielding effect of Na^+ and Cl^- ions on the dye molecules.

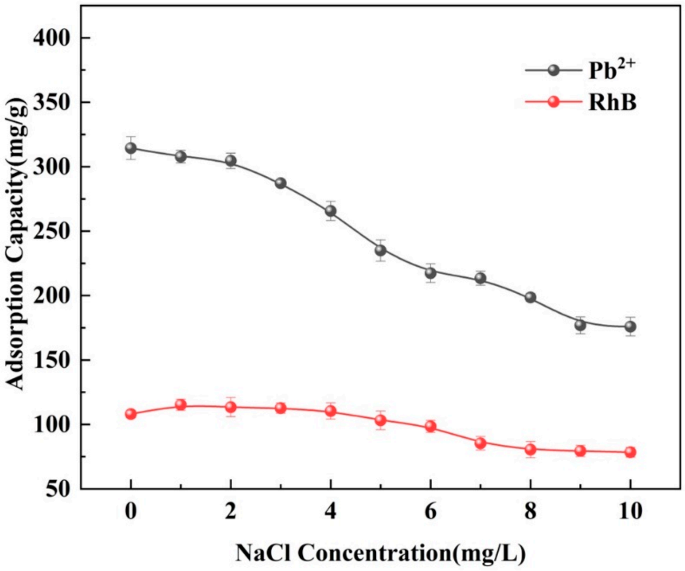

Figure S3. The effect of ion strength on the adsorption capacities of GP/CTS/AA-co-AM for Pb^{2+} and RhB.

8. Zeta Potential

Figure S4 presents the Zeta potential of the adsorbents GP/CTS/AA-co-AM across various pH levels. Within the pH range of 2 to 12, the surface charge of the hydrogel exhibited a negative polarity. As the pH increased, a gradual reduction in the surface charge was observed, ranging from –0.32 mV to –24.93, facilitating enhanced adsorption of contaminants.

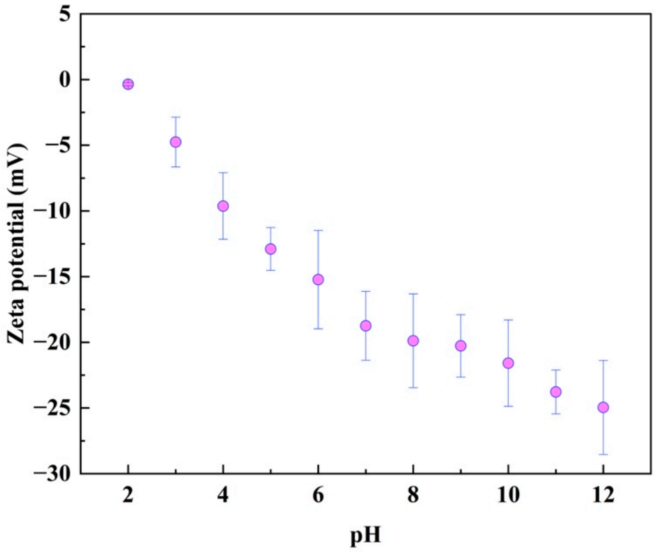

Figure S4. The zeta potential of GP/CTS/AA-co-AM at different pH.

9. Thermodynamic studies

Thermodynamic parameters were calculated using:

$$\Delta G^{\circ} = -RT\ ln\, k_{\mathrm{L}} \quad (1)$$

$$ln\, k_{\mathrm{L}} = \frac{\Delta S^{\circ}}{R} - \frac{\Delta H^{\circ}}{RT} \quad (2)$$

Where, R is the universal gas constant (8.314×10^{-3} kJ/mol·K) and T is the temperature (K), k_L is the Langmuir adsorption equilibrium constant (L/mg).

Table S6. Thermodynamic parameters of LR-EPI-8 for Pb(II) and Cu(II).

Gel	T (K)	Pollutants	ΔGº(kJ/mol)	ΔSº(J/mol·K)	ΔHº(kJ/mol)
GP/CTS/AA-co-AM	298	Pb(II)	– 4.36	46.26	0.67
	308		–6.22		
	318		– 4.05		
	298	RhB	– 7.36	43.84	1.34
	308		– 8.18		
	318		– 7.04		

Illustrated in Figure S5, the adsorption capacity demonstrated a slight increase with the elevation of temperature from 298 K (25°C) to 308 K (35°C). This phenomenon can be attributed to the swelling of hydrogels and a reduction in the viscosity of the solution. However, a subsequent decrease in adsorption capacity was noted as the temperature exceeded the range of 308 to 318 K (45°C). This decline can be rationalized by the superior diffusion rate of contaminants ion and molecules.

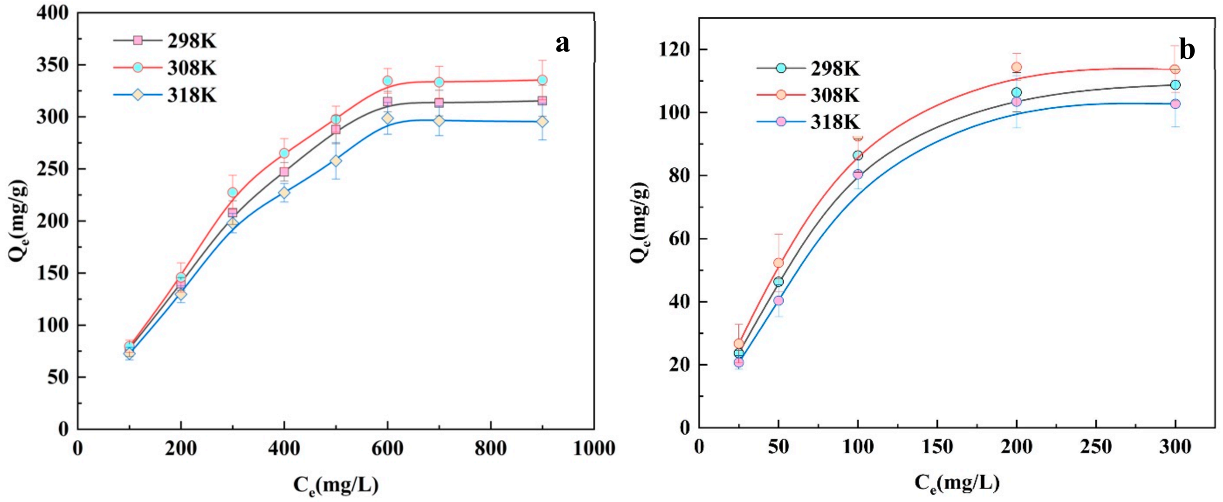

Figure S5. Adsorption thermodynamic for adsorption of Pb^{2+}(a) and RhB (b)on GP/CTS/AA-co-AM.

Reference

[1] Liu, S., Huang, J., Zhang, W., Shi, L., Yi, K., Zhang, C., Pang, H., Li, J., & Li, S. Investigation of the adsorption behavior of Pb (II) onto natural-aged microplastics as affected by salt ions. J. Hazard. Mater., 2022, 431, 128643.
